# Ice nucleation imaged with X-ray spectro-microscopy

**DOI:** 10.1039/d1ea00077b

**Published:** 2022-02-07

**Authors:** Peter A. Alpert, Anthony Boucly, Shuo Yang, Huanyu Yang, Kevin Kilchhofer, Zhaochu Luo, Celestino Padeste, Simone Finizio, Markus Ammann, Benjamin Watts

**Affiliations:** Laboratory of Environmental Chemistry, Paul Scherrer Institute 5232 Villigen PSI Switzerland Peter.Alpert@psi.ch; Electrochemistry Laboratory, Paul Scherrer Institute 5232 Villigen PSI Switzerland; State Key Joint Laboratory of Environment Simulation and Pollution Control, School of Environment, State Environmental Protection Key Laboratory of Sources and Control of Air Pollution Complex, Beijing Key Laboratory of Indoor Air Quality Evaluation and Control, Tsinghua University Beijing 100084 China; Laboratory for Multiscale Materials Experiments, Paul Scherrer Institute 5232 Villigen PSI Switzerland; Laboratory for Mesoscopic Systems, Department of Materials, ETH Zürich Zürich Switzerland; Laboratory of Nanoscale Biology, Paul Scherrer Institute 5232 Villigen PSI Switzerland; Swiss Light Source, Paul Scherrer Institute 5232 Villigen PSI Switzerland

## Abstract

Ice nucleation is one of the most uncertain microphysical processes, as it occurs in various ways and on many types of particles. To overcome this challenge, we present a heterogeneous ice nucleation study on deposition ice nucleation and immersion freezing in a novel cryogenic X-ray experiment with the capability to spectroscopically probe individual ice nucleating and non-ice nucleating particles. Mineral dust type particles composed of either ferrihydrite or feldspar were used and mixed with organic matter of either citric acid or xanthan gum. We observed *in situ* ice nucleation using scanning transmission X-ray microscopy (STXM) and identified unique organic carbon functionalities and iron oxidation state using near-edge X-ray absorption fine structure (NEXAFS) spectroscopy in the new *in situ* environmental ice cell, termed the ice nucleation X-ray cell (INXCell). Deposition ice nucleation of ferrihydrite occurred at a relative humidity with respect to ice, *RH*_i_, between ∼120–138% and temperatures, *T* ∼ 232 K. However, we also observed water uptake on ferrihydrite at the same *T* when deposition ice nucleation did not occur. Although, immersion freezing of ferrihydrite both in pure water droplets and in aqueous citric acid occurred at or slightly below conditions for homogeneous freezing, *i.e.* the effect of ferrihydrite particles acting as a heterogeneous ice nucleus for immersion freezing was small. Microcline K-rich feldspar mixed with xanthan gum was also used in INXCell experiments. Deposition ice nucleation occurred at conditions when xanthan gum was expected to be highly viscous (glassy). At less viscous conditions, immersion freezing was observed. We extended a model for heterogeneous and homogeneous ice nucleation, named the stochastic freezing model (SFM). It was used to quantify heterogeneous ice nucleation rate coefficients, mimic the competition between homogeneous ice nucleation; water uptake; deposition ice nucleation and immersion freezing, and predict the *T* and *RH*_i_ at which ice was observed. The importance of ferrihydrite to act as a heterogeneous ice nucleating particle in the atmosphere using the SFM is discussed.

## Abstract 2

Ice nucleation can now be imaged *in situ* using X-ray spectro-microscopy in a new experiment, which is applied to mineral aerosol particles composed of ferrihydrite or feldspar and associated organic matter.

## Graphical abstract

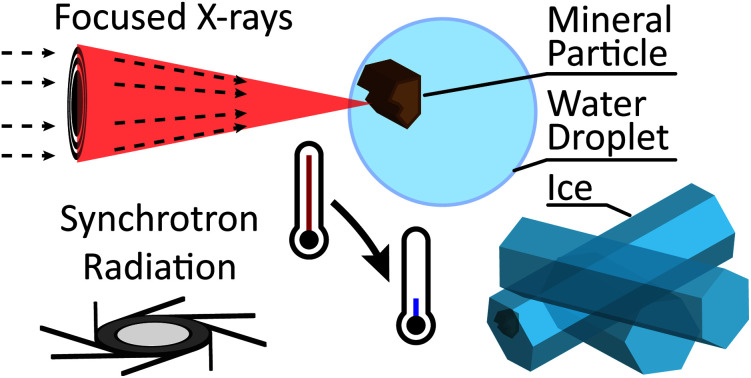

**Environmental significance**

Snow, hail, and about two-thirds of rain begins as ice crystals. Therefore, the initial formation of ice crystals, or ice nucleation, in the upper atmosphere is a critical step in the Earth's water cycle. Particles present in the air or within cloud droplets can catalyze ice nucleation in what is called heterogeneous ice nucleation. We studied this with a range of particle types in a newly developed cryogenic environmental cell that allows nano-focused X-ray experiments to be performed while ice nucleation is occurring. We have found that particles of an important mineral in airborne dust, ferrihydrite, aids ice nucleation and so is important for cloud formation. When the concentrations of such particle types are known, our work can be used to accurately predict the amount of ice formed for better representation of clouds in weather and climate models.

## 1 Introduction

Ice formation in the atmosphere is important for accurate representation of the hydrological cycle,^1–3^ of stratospheric water vapor,^4,5^ of multi-phase chemical reactions on ice,^6–9^ and thus for climate projection overall.^10–12^ Furthermore, ice plays a large role in precipitation, not only for snow, sleet and hail, but also for rain – about two-thirds of rain begins as ice crystals.^13^ In mixed-phase clouds where ice particles and liquid droplets co-exist, ice crystals grow and remove water vapor by sedimentation, which can affect cloud lifetime and reflectivity of solar radiation.^5^ Cirrus clouds are composed entirely of ice crystals and on average, they have a warming effect (positive radiative forcing) due to them being fairly transparent to solar radiation and strongly absorbing terrestrial radiation.^14,15^ Aerosol particles are playing a key role in the formation of ice. They have varied composition and properties, including organic and inorganic matter, as well as water soluble and insoluble components.^16–18^ This has led to a wide range of thermodynamic conditions over which they can nucleate ice. Aerosol-cloud interactions can influence Earth's radiation balance, atmospheric temperature, and cloud evolution.^5,11,19–21^ However, accurate representation of aerosol–ice cloud interactions and any resulting climatic effects requires a significant reduction in the uncertainties of quantifying how aerosol particles influence ice cloud abundance, albedo, height, and especially overall ice particle numbers in clouds.^5,21^

Predicting atmospheric ice cloud formation from aerosol particles is difficult, partly because ice can form in different ways depending on the aerosol composition and phase.^20,21^ Homogeneous ice nucleation can occur from micrometer sized liquid cloud droplets and aqueous aerosol particles below about 235 K.^22–25^ Heterogeneous ice nucleation in the atmosphere can occur from the liquid or the vapor phase of water on aerosol particles, termed ice nucleating particles (INPs). Furthermore, the ability to form ice can be highly variable between different substrates including insoluble particles, crystalline particles, biogenic particles or macromolecules, compressed surfactants on droplets and highly viscous (or glass-like) solid aerosol particles.^20,23,26–28^ Atmospheric particles experience continuously changing relative humidity with respect to water, *RH*_w_, and temperature, *T*, for a wide range of water vapor pressures. Therefore, knowing the precise *RH*_w_ and *T* that define which phase transitions to ice are dominant is of great value. Immersion freezing (liquid-to-ice) is a heterogeneous ice nucleation mode that describes liquid water in an aqueous aerosol or cloud droplet initiating freezing on a particle surface immersed within the liquid. Another heterogeneous ice nucleation mode is deposition ice nucleation (vapor-to-ice), whereby water molecules from the supersaturated vapor phase nucleate ice and subsequently form an ice crystal on a particle surface without the involvement of a bulk liquid aqueous phase. Other freezing modes include contact freezing, condensation freezing, pore-condensation freezing or inside-out freezing,^29–32^ however these are not considered here. Surfactant substrates can nucleate ice heterogeneously,^33–37^ although, the impact of ambient organic surfactants on ice formation in atmospheric clouds is fairly understudied compared with laboratory based investigations.^38^ In particular, the kinetics of ice nucleation for immersion freezing have been quantified using a universal approach based on aqueous solution water activity in laboratory and field studies applicable for a variety of substrates previously mentioned including mineral, organic, biological, surfactant, and soot.^39–42^ Use of water activity in this study to predict freezing is detailed in a later section. It is important to note that an ice nucleating substrate can be solid-like (glassy or highly viscous), but not necessarily water insoluble.^20,43–45^ In this study, we have aimed to investigate whether deposition ice nucleation, immersion freezing or homogeneous ice nucleation is favored over the other depending on the particle types present.

Iron containing aerosol particles are a subset of mineral dust particles that are abundant^46^ and found in the residuals of ice crystals.^47–50^ Laboratory and model studies have revealed that common minerals and clay such as feldspar, a highly important atmospheric ice nucleating particle, kaolinite and other iron containing minerals are ice forming particles.^40,51–59^ Iron is frequently incorporated into airborne clay mineral particles and can be at about 5 weight percent as inclusions of iron oxy-hydroxides.^60^ Additionally, iron mass abundance is typically equal to that of calcium and aluminum in fine dust.^46^ Anthropogenic iron from urban and combustion sources can be present in about 1 in 20 particles^61^ and make up a high fraction of soluble iron globally, suspected to be due to an abundance of ferrihydrite.^62^ Ferrihydrite, or colloidal iron oxy-hydroxides, is an amorphous mineral phase responsible for the transition between soluble iron (*i.e.* iron ions) and hematite or goethite.^63^ Iron minerals such as hematite and goethite are heterogeneous ice nucleators and their freezing efficiency increases (*i.e.* freezing can occur at warmer temperatures) when milled or when particle surface area is increased by other chemical or physical treatments.^64,65^ The authors of these studies explain their results with milled and unmilled particles by correlating the freezing temperatures with differences in OH group surface concentration and arrangement, in addition to the degree of ice crystal lattice-match.^64,65^

Ferrihydrite is an amorphous iron oxy-hydroxide. In general, iron oxy-hydroxides amount to more than half of all iron in Asian and African dust aerosol particles,^66,67^ and to a quarter of iron containing particles from oil combustion.^66^ Although it is thermodynamically unstable,^68,69^ ferrihydrite is ubiquitous in nature^70^ and can remain untransformed for a few hundred days in the atmosphere.^71^ This long lifetime has been reportedly due to silica, clay minerals, and a range of organic substances (all present in Saharan soils and dust) that contribute to its stabilization.^72^ Importantly, ferrihydrite is also considered to be a product from the weathering process of iron containing clays such as illite or smectite^69^ and indeed present at the surface of such clay minerals.^69,73^ Unlike goethite and hematite, ferrihydrite has no clear structure and previously observed to be in the form of nanoaggregates.^63,74,75^ It exists in two types, exhibits 2 or 6 lines in X-ray diffractograms and has no fixed composition.^63^ Due to its porous nanostructure, ferrihydrite has a high specific surface area and offers a high number of accessible sites for adsorption and reaction. It is also considered to be the main source of bio-available iron, *i.e.* leached from the mineral and absorbed by organisms and thus is critically important to marine life.^63,74,75^ Thus, ferrihydrite can significantly impact atmospheric chemistry, iron fertilization in oceans, and in particular, ice cloud formation. Although, its capability to nucleate ice has not previously been identified, likely due to the difficulty in isolating it from ambient air and from other minerals, as well as being difficult to synthesize in the laboratory. Being quite unstable, ferrihydrite is also difficult to accurately identify and characterize in laboratory samples.

Here, we have used a unique pairing between scanning transmission X-ray microscopy coupled to near-edge X-ray absorption fine structure spectroscopy (STXM/NEXAFS) and a new ice nucleation experiment, termed the INXCell, to simultaneously observe the formation of ice in the vicinity of specific particles under tightly controlled temperature and humidity conditions. The composition and chemical state of particles (those that did and did not nucleate ice) were imaged *in situ* with stability and control of *RH*_w_ and *RH*_i_, which was not previously achievable in a STXM experiment. Environmental scanning electron microscopy (ESEM) has previously been used for imaging ice nucleation *in situ*.^76–78^ Although spatial resolution of SEM is superior to STXM, energy-dispersive X-ray spectroscopy used with SEM has inferior chemical selectivity compared with STXM/NEXAFS.^79,80^ Our method also does not require sample transport or vacuum exposure in between ice nucleation observations and chemical investigation as in offline studies,^48,50^ eliminating sudden exposure of particles to vast swings of *T* and *RH*_w_. STXM/NEXAFS requires thin (∼100 nm) and delicate silicon nitride windows for an X-ray transparent support for the particles, however this results in very poor survivability of samples for offline studies due to transport, handling and preparation. Our INXCell also facilitates easier identification of single ice nucleating particles. This avoids the difficulty for offline studies due to the differences in orientation, contrast generation and spatial resolution between various microscopes, *e.g.*, optical and X-ray, that can hinder re-identification of particles. These difficulties are evident due the fact that only one particle observed to nucleate ice was ever observed using STXM/NEXAFS offline,^50^ and ice was only tentatively observed in another X-ray environmental cell without knowledge of the nucleating *T* or *RH*_i_ conditions.^81^ When considering ice nucleation capability, spatial resolution and chemical selectivity, the INXCell is superior to other techniques, *e.g.* Raman spectro-microscopy, typically having a 1 × 1 μm^2^ pixel size.^82^ This is important when investigating submicrometer sized particles or ambient particles that can be highly diverse.

We quantify the ice nucleation ability of bare ferrihydrite, ferrihydrite mixed with citric acid, and K-rich microcline feldspar mixed with xanthan gum. Citric acid was used as a proxy for oxygenated organic material common in atmospheric aerosol particles and has a similar viscosity behavior.^83,84^ Xanthan gum is a proxy for polymeric or oligomeric compounds common in atmospheric organic particles^85,86^ and primary marine organic matter.^87,88^ The glass transition of xanthan gum was previously documented,^89^ and beneficial to explain the impact on ice nucleation mode when particles are more solid or liquid-like. The INXCell encloses a volume between two X-ray transparent membranes where the local gaseous environment is provided from a controlled external flow and the temperature is controlled by blowing cold nitrogen gas against the membrane supporting the test particles. A platinum wire sensor printed onto the membrane was used to measure the temperature with high precision and accuracy. The pair of membranes provide a window through which the sample volume of INXCell can be observed *in situ* with STXM/NEXAFS spectroscopy.^79,80,90^ This allows the composition and chemical state of particles to be mapped *in situ*, as well as having the stability and control to image particles inside ice crystals and water droplets at saturation with respect to liquid or ice (*RH*_w_ = 100% or *RH*_i_ = 100%, respectively). Additionally, we have advanced a previous model for homogeneous ice nucleation and heterogeneous ice nucleation from the vapor phase to complement our experimental results that builds on our previous work.^40^ This model is designed to derive heterogeneous ice nucleation rates with carefully estimated uncertainties. Together, our new method and model makes it possible to investigate single ice nucleating particles *in situ* as a function of *T*, *RH*_w_, and *RH*_i_.

## 2 Experimental

The INXCell is based on STXM/NEXAFS,^79,80,90^ traditional cryogenic cold-stage optical microscopy ice nucleation techniques,^91,92^ and in general, water uptake and ice formation are imaged and spectroscopically probed.^81,93–100^ Controlling the environmental conditions with enough precision to navigate the phase changes while also keeping the sample area thin enough for transparency of the soft X-ray beam is very challenging, as illustrated by the fact that only one study has ever tentatively detected ice under controlled conditions,^81^ and that pure water droplets formed from the vapor phase have never before been observed using STXM/NEXAFS. A key feature in the design of the INXCell was the use of lithographic patterning to fabricate high precision temperature sensors directly onto sample substrates, placing the sensor directly in the measurement region and allowing precise determination of the thermodynamic conditions when ice nucleates on particles within the INXCell. Previous *in situ* ice nucleation studies with higher spatial resolution than optical microscopy have utilized environmental-scanning electron microscopy (E-SEM), in which a sample is imaged while resting on a thick, cold, and thermally conductive block in a humidified atmosphere.^51,76–78,101^ These studies have advanced our understanding of how morphological features of individual particles or surfaces can impact freezing and crystallization. However, E-SEM does not have chemical sensitivity, compared to STXM/NEXAFS, which can identify organic coatings and provide information on chemical bonding and metal speciation within individual particles. This is important due to the great chemical complexity that ambient INPs can have.^50,78^ Both INPs^50^ and the residuals of ice crystals^48^ have been probed using X-ray spectro-microscopy in offline analysis. In terms of *in situ* analysis, previous studies have successfully produced X-ray transparent, layered structures for the purpose of investigating time resolved changes in magnetic domains,^102–105^ electrochemical investigation^106^ and calorimetric studies.^107,108^*In situ* microreactors have been previously developed and used in the field of atmospheric chemistry and physics for investigating water uptake and phase transformations,^81,93–97^ and chemical^98,99^ and photochemical reaction cycling.^100,109^

### 2.1 INXCell design and description

**Fig. 1:**
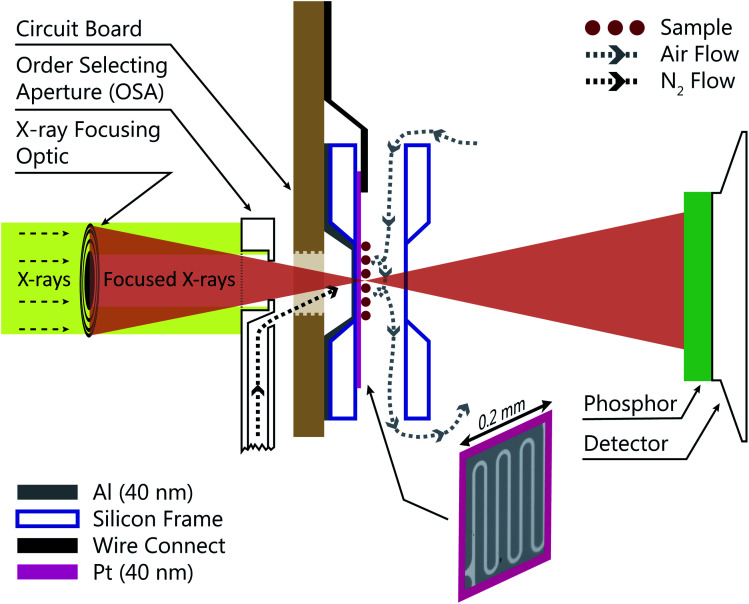
Sketch of the INXCell configuration. X-rays were focused by a Fresnel zone plate through a temperature controlled order selecting aperture (OSA) with an integrated gas jet. Particles inside the INXCell were exposed to humidified air and cooled by the impinging gas jet from the OSA. The INXCell was mounted on a circuit board (light brown) that was connected to a lithographically fabricated temperature sensor on the sample substrate with a thickness of 40 nm. An optical microscope image of the sensor is outlined in pink. Transmitted X-rays were detected using a phosphor screen coupled with a photomultiplier tube (PMT). Further details are given in the text. When desired, a 40 nm aluminum layer was condensed onto the reverse side (upstream facing) of the membrane to increase the lateral thermal conductivity. The flows of humidified sample air and dry nitrogen is depicted. The sketch is not drawn to scale.

Fig. 1 shows a schematic of the INXCell that highlights the two most important design features. The first is a patterned temperature sensor on the surface of the silicon nitride membrane. An image of the sensor is outlined in pink in Fig. 1 and is at the exact position of the particles on which ice nucleates. The sensor is a 40 nm thick and 10 μm wide platinum wire fabricated using standard electron beam lithography (VISTEC EBPG 5000Plus)^102,105^ and inspired by a previous study.^108^ Briefly, a 40 nm thick film of platinum was deposited with a serpentine pattern in a PMMA/MMA (poly methyl methacrylate/methyl methacrylate) bilayer resist prepared by electron beam lithography with a direct current magnetron sputter coater (AJA) at a base pressure <2 × 10^−8^ torr and deposition pressure of 3 mTorr. The platinum and resist were on a 1 mm square silicon nitride window having a 100 nm membrane thickness supported by a 0.2 mm thick silicon frame that was 5 mm square. The exposed resist was developed by immersion in a 1 : 3 by volume mixture of methyl isobutyl ketone and isopropyl alcohol and rinsed in pure isopropyl alcohol. A lift-off step was performed by immersing the patterned membrane in pure acetone to remove the resist and extraneous platinum over layer, but retaining the platinum wire pattern. Contacts extended from the membrane to the supporting silicon frame and were wire bonded (TPT HB05) to a custom printed circuit board^102,105^ connected to a high precision electrometer (Keithley Series 4200). The sensor resistance was around 600 Ω at room temperature and a sample specific temperature calibration, described in a later section, was performed.

The second important design consideration was to ensure that the sample of particles on the silicon nitride membrane was the coldest surface to prevent icing in undesirable locations of the INXCell construction. The requirement for X-ray transparency limits the thickness of the membranes enclosing the cell and supporting the sample particles to about 0.1 μm. This means that the membrane transports heat easily through the thickness of the membrane, but very poorly across its much larger width,^108^ and so the sample particles near the center of the membrane cannot be efficiently cooled by the cell body. Therefore, an additional form of cooling must be applied from a direction out of the plane of the membrane. This is further hindered by the space restriction of the STXM optics; the Fresnel zone plate (X-ray diffraction focusing optic) requires an order selecting aperture (OSA) positioned within a few hundred micrometers of the focus in order to block the unfocused, zero-order light, while allowing the first-order focus to pass through, as illustrated in Fig. 1. Therefore, we developed a temperature controlled OSA with a gas outlet that delivers a jet of cooled N_2_ to the front of the cell, removing heat from the center of the membrane and then being pumped from the experiment chamber by a turbomolecular pump. In some instances, a 40 nm layer of Al was deposited on the reverse side of the sample membrane to marginally increase thermal conductivity across its surface. The total pressure inside the cell was maintained at 150 mbar and the vacuum chamber pressure was ∼10^−3^ mbar. Finally, the body of the INXCell was also cooled and temperature controlled at about 1–2 K warmer than the OSA. It is this two-step cooling design, *i.e.* cooling of the INXCell body and further cooling of the sample surface utilizing the OSA, resulted in the sample position having the lowest temperature in the gas flow system and being the only area for ice nucleation to occur.

The INXCell chamber dimensions and airflow are identical to previous designs,^93,98,99^ with minor improvements such as thermally insulating the mounting plate attached from below. Dry helium was used as the carrier gas inside the cell at 20 cm^3^ min^−1^ (at equivalent standard temperature and pressure). First, all residual water vapor was removed from the He flow using a liquid nitrogen trap. The flow was split and directed to either a humidifier or a bypass. The temperature controlled humidifier was half-filled with ice held at −16 °C and separated from the air above using a layer of Nafion film. The humidified flow was mixed with the dry bypass flow to achieve a controlled frost point temperature down to about 215 K. Pressure was measured just before the inlet gas flow line of the INXCell and controlled at 150 mbar using a vacuum pump and a proportional-integral-derivative (PID) controlled solenoid valve. Outside of the INXCell in the STXM/NEXAFS vacuum chamber, a 100 cm^3^ min^−1^ flow of N_2_ was directed through the cooled OSA onto the reverse side of the sample substrate. This resulted in a vacuum chamber pressure of ∼10^−3^ mbar, sufficiently low to minimize the X-ray absorption outside of the gas cell. The N_2_ flow was pre-dried in the same water vapor trap as for the He flow with an additional pressure regulator.

### 2.2 Ice nucleation and humidity calibration procedure

The temperature of the INXCell, *T*_cell_, and of the OSA supporting arm, *T*_OSA_, was measured with calibrated Pt-100 sensors and controlled using PID modules. Prior to starting any ice nucleation experiments, a temperature calibration of each lithographically patterned Pt sample sensor was performed. To do this, the sample resistance was measured while dry gas was flowing through the INXCell and OSA. The position of the INXCell and OSA was the same as when obtaining a focused STXM image. Then, the OSA and INXCell were cooled to the same *T*, *i.e. T*_OSA_ = *T*_cell_ and the resistance of the Pt sample sensor, *R*_p_, was measured. Therefore, the temperature of the particles on the membrane, *T*_p_, was equal to *T*_OSA_ and *T*_cell_, and corresponded to *R*_p_. This was repeated every 10 K over the range 210–290 K to derive a temperature-resistance calibration curve.

To conduct an ice nucleation experiment, *T*_OSA_ and *T*_cell_ were initially set to the same temperature and *RH*_i_ was set to just under <90%. When a cooling ramp began, *T*_cell_ was fixed and *T*_OSA_ was cooled at a rate of 0.3 K min^−1^, which led to a change in *RH*_i_ of about 2.8% min^−1^ and similar to other methods.^92,110^ During cooling, we continuously imaged the sample area at a single X-ray energy until ice formed. Ice crystals grew to hundreds of micrometers in size over a few minutes and were clearly identifiable in the STXM images as areas of dark contrast. As soon as ice was detected, *T*_OSA_ was immediately increased to ∼ 1–3 K warmer than the temperature at which ice nucleated in attempt to stop crystal growth. Water uptake was identified when imaged particles instantaneously and clearly increased in size and X-ray absorption while being cooled. It is notable that these effects were much less pronounced than ice crystal appearance and growth^92,110^ (as seen in Fig. 4(a) and discussed in a later section).

Calibration of *RH*_i_ was performed after each observation of ice nucleation following previous optical microscopy studies.^91,92^ Briefly, STXM images of ice crystals increasing or decreasing in size indicated when *RH*_i_ was supersaturated (>100%) or subsaturated (<100%), respectively. We determined *T*_p_ at which an ice crystal did not change size over multiple images, *i.e.* for a time of ∼10 min. The measured temperature at which ice crystals did not change size is the so-called frost point temperature, *T*_fst_. Then, we calculated 
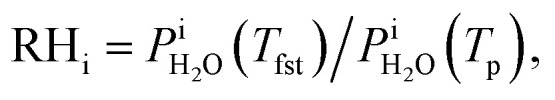
 where 
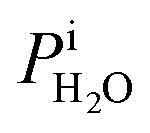
 is the saturation vapor pressure over ice as a function of temperature. In some instances, ice nucleation did not occur and instead, only water droplets formed. Then, 
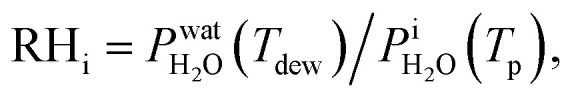
 where *T*_dew_, is the so-called dew point temperature. The same calibration procedure was implemented except determining the temperature at which water droplets did not grow or shrink, equivalent to *T*_dew_. After calibration, ice crystals were sublimated while continuously being imaged. The residual particles remaining beneath the sublimated crystals were then investigated using the highest spatial and X-ray energy resolution possible. The error on resistance was <±0.3 Ω, which translated to <±0.2 K in *T*. The uncertainty in both *T*_dew_ and *T*_fst_ was then chosen as ±0.2 K. This was propagated to 
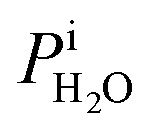
 or 
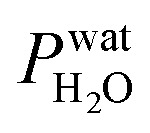
 and then to the error in *RH*_i_ found in Table 1. Note, the time between each X-ray image acquisition was about 40 s, corresponding to a systematic uncertainty range in *RH*_i_ of about 2%, which is far less than the error given in Table 1. Cooling cycles were repeated *N* times given in Table 1. Before each cycle, particle temperature was increased to 275 K to prevent any effects from preactivation.^92,111^

**Table 1:** The relative humidity with respect to ice, *RH*_i_, and its uncertainty, *σ*_*RH*i_, temperature, *T* and the water activity criterion, Δ*a*_w_, at which ice nucleated on average for *N* freezing cycles having a frozen fraction of particles, *f*, surface area, *A*, heterogeneous ice nucleation rate coefficient, *J*_het_, and the ice crystal count normalized to *A*, *n*_s_

Name	*T*/K	*RH*_i_	*σ*_*RH*i_	Δ*a*_w_	*N*	*f*	*A*	*J*_het_/cm^−2^ s^−1^	*n*_s_/cm^−2^
FH_Dep_^a^	231.7	131.3	±6.6	0.210	7	2 × 10^−2^	2.0 × 10^−6^	4.4 × 10^2^	2.6 × 10^4^
FH_Im_^b^	231.4	145.0	±6.7	0.301	1	2 × 10^−2^	2.0 × 10^−6^	2.7 × 10^2^	1.6 × 10^4^
FH_Im_	234.9	138.5	±6.2	0.266	3	2 × 10^−2^	2.0 × 10^−6^	8.2 × 10^1^	4.9 × 10^3^
FH_WU_^c^	231.6	141.5	±6.6	0.278	1	—	2.0 × 10^−6^	—	—
FH_WU_	235.7	127.4	±5.7	0.191	3	—	2.0 × 10^−6^	—	—
FH_WU_	240.0	138.2	±6.0	0.277	1	—	2.0 × 10^−6^	—	—
FH + CA_Im_^d^	230.1	150.3	±7.0	0.332	1	1 × 10^−5^	1 × 10^−4^	8.0 × 10^2^	4.8 × 10^4^
FH + CA_Im_	238.1	140.7	±6.2	0.289	2	1 × 10^−5^	1 × 10^−4^	1.8 × 10^2^	1.1 × 10^4^
FsXG_Dep_^e^	243.9	119.0	±6.4	0.143	4	6 × 10^−3^	9.6 × 10^−6^	4.6 × 10^2^	2.8 × 10^4^
FsXG_Im_^f^	255.3	113.9	±6.2	0.117	2	6 × 10^−3^	9.6 × 10^−6^	2.5 × 10^1^	1.5 × 10^3^
FsXG_WU_^g^	255.8	108.3	±4.1	0.070	2	—	9.6 × 10^−6^	—	—

a Deposition ice nucleation on ferrihydrite.

b Immersion freezing after water uptake on ferrihydrite.

c Water uptake on ferrihydrite.

d Immersion freezing after water uptake on ferrihydrite/citric acid particles.

e Deposition ice nucleation on feldspar/xanthan gum particles.

f Immersion freezing after water uptake on feldspar/xanthan gum particles.

g Water uptake on feldspar/xanthan gum particles.

### 2.3 Particle preparation

Ferrihydrite particles were dry deposited on the silicon nitride membrane. Briefly, ferrihydrite synthesis followed an adapted version of Schwertmann's method,^112^ where 100 ml of Fe(NO_3_)_3_ at 0.1 M was prepared in a beaker and titrated by 0.1 M of NaOH under strong stirring until the pH of the solution reached 7.5–8. The product in the form of precipitate was then washed six times with twice its volume of distilled water through a centrifugation procedure. Finally, the product was dried in an oven for 48 h at 40 °C.^112^ When desired, aqueous citric acid droplets were deposited on top first by nebulizing a 3 weight percent solution, and impacting them on top of the ferrihydrite particles. The solution droplets were generated using an ultrasonic nebulizer.^98,99^ K-rich feldspar particles mixed with xanthan gum were also dry deposited from a powder. Feldspar was purchased from the Bureau of Analysed Sample Ltd (BCS-CRM no. 376/1 SGT FELDSPAR 1) and sieved through a 250 μm mesh. This was milled again with a ball-milling machine for 5 min and sieved through a 64 μm mesh. A bulk sample was prepared by mixing a small volume of xanthan gum, feldspar and water in equal weight ratio. After drying for about two weeks, the mixture turned to a glass state, which was then crushed into a powder using a mortar and pestle. The nebulizer was cleaned before each use, first by scrubbing using laboratory detergent, then rinsing in distilled and deionized water (resistivity of 18.2 MΩ) and third, soaking in 10% weight percent HCl solution for one hour followed by another rinse prior to use. Tubing and pumps that supplied liquid to the nebulizer were cleaned by circulating the HCl solution for one hour, followed by a rinsing with distilled and deionized water. The mortar and pestle were cleaned following the same procedure. This ensured that undesired compounds were below detection, which was confirmed by generating sodium chloride particles after a cleaning procedure without detecting iron or carbon using STXM/NEXAFS. Confirmation of coatings on mineral particles was performed using STXM/NEXAFS where particles were imaged at the carbon pre-edge at 280.0 eV and either the peak absorption for citric acid at 288.5 eV or xanthan gum at 289.4 eV. X-ray absorption increased uniformly over the particles and appeared slightly enlarged indicating a complete coating. However, STXM images are 2-D and thus we cannot exclude the possibility that some bare feldspar was exposed at the top of the particles. However, we find this unlikely and maintain the presumption that feldspar was completely coated. Future studies should take care to quantify coating amount and heterogeneity. Multiple images of particles were used to derive 2-D projected surface area, which were then translated to 3-D particle surface area estimates assuming spherical geometry. The particle surface area per field of view from multiple images was scaled up to the total sample area estimated to be about 0.16 mm^2^. Due to time constraints using the synchrotron X-ray beamline, our experiments were limited to the investigation of ferrihydrite particles, ferrihydrite particles coated with citric acid and feldspar coated with xanthan gum. We suggest further experiments on ferrihydrite particles coated with xanthan gum and feldspar particles without any coatings as an addition to the current dataset.

### 2.4 STXM/NEXAFS procedure

The INXCell was developed and operated at the PolLux beamline of the Swiss Light Source using STXM/NEXAFS.^79^ X-rays were first focused using a Fresnel zone plate to a minimum pixel size of either 50 or 35 nm at the position of the sample, depending on the specific zone plate used. The custom designed OSA was positioned between the zone plate and sample to block unfocused and unwanted light. The transmission of X-ray photons through the particle was measured and converted to optical density, *OD* = − ln(*I*/*I*_0_), where *I*_0_ and *I* are the incident and transmitted photon count, respectively, and with an uncertainty of 
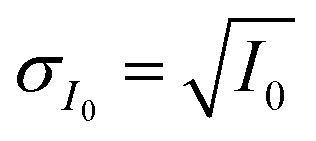
 and 
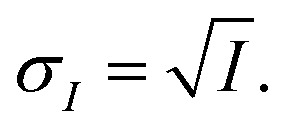
 The sample was scanned over a desired field of view (FOV), while the focused X-rays remained at a fixed position. An image was acquired from hundreds to thousands of individual pixels, where *OD* was calculated for each pixel. Multiple *OD* images over the same FOV taken over a range of X-ray energies, referred to here as a “stack”, were aligned and processed using publicly available software.^113^ We primarily investigated the X-ray energy ranges between 278–320 eV, 525–550 eV and 700–735 eV, which correspond to the carbon K-edge, oxygen K-edge and iron L_2,3_-edges, respectively.

## 3 Results and discussion

### 3.1 Observation of ice nucleation

**Fig. 2:**
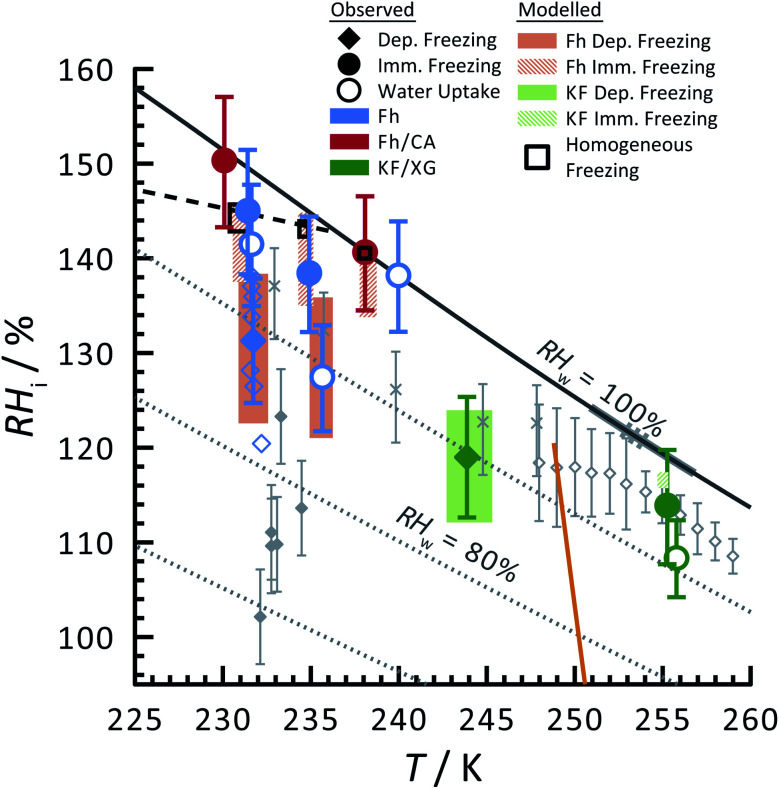
Ice nucleation observations from ferrihydrite, mixed ferrihydrite and citric acid, and mixed K-feldspar and xanthan gum particles as a function of relative humidity with respect to ice, *RH*_i_, and temperature, *T*. Deposition ice nucleation, immersion freezing and water uptake are indicated by different symbols given in the legend, where each are averages of repeat experiments. An example of individual data points are shown as small open blue diamonds for ferrihydrite deposition ice nucleation. Solid and hashed orange boxes indicate the modelled range for deposition and immersion freezing, respectively, from ferrihydrite. Solid and hashed light green boxes indicate deposition ice nucleation and immersion freezing, respectively, from feldspar. Black open boxes are the modelled range of homogeneous freezing. Grey filled diamonds are freezing due to hematite,^64^ crosses are freezing of K-feldspar particles with an electrical mobility diameter of 300 nm,^58^ and gray open diamonds are deposition ice nucleation of K-feldspar particles with diameters between 1–100 μm,^51^ respectively. The gray solid^57^ and dashed^55^ lines are a range of immersion freezing of K-feldspar in water droplets. The orange line is the expected glass transition temperature for aqueous xanthan gum solutions.^89^ The solid black line indicates water saturation, *i.e.* when the relative humidity with respect to water, *RH*_w_ = 100%. Dotted gray lines indicate decreasing *RH*_w_ by 10%. The black dashed line indicates homogeneous ice nucleation from a single water or aqueous solution droplet ∼ 10 μm in diameter.

Ferrihydrite particles are effective heterogeneous INPs. Ice nucleation was observed as a function of *T* and *RH*_i_ and shown in Fig. 2. Deposition ice nucleation was observed (when liquid water was not detected) at about 232 K and *RH*_i_ = 132% seen as the filled blue diamond, which is the average of multiple freezing cycles. Individual *RH*_i_ and *T* values for each cycle are shown as small blue open diamonds. Water uptake (open circles) for ferrihydrite particles was observed for only one cycle at *T* = 231 K, while it was always observed when *T* = 236 K. Note that water condensation was observed at subsaturated conditions for these temperatures, *i.e. RH*_w_ < 100%. After water uptake, ice nucleation occurred (filled circles) at or slightly below conditions expected for homogeneous ice nucleation. This result implies that there was a competition between deposition ice nucleation and water uptake. At *T* = 240 K, ice nucleation was not observed, and water uptake occurred close to *RH*_w_ = 100%. When ferrihydrite was present with citric acid, ice nucleation was observed at or slightly below conditions for homogeneous freezing when *T* = 230 K. Water uptake due to citric acid already occurred when *RH*_i_ < 90% and was not quantified in this study. Additional information about freezing results and particle sample properties are found in Table 1. We compare our results with those of hematite^64^ having a similar sample surface area shown as small filled diamonds. Note, that these data points are onset conditions, *i.e.* the *RH*_i_ and *T* at which ice and water uptake was first observed. Ice nucleation on hematite occurred at considerably less *RH*_i_ with roughly the same scatter in the data. Clearly, ice nucleation for ferrihydrite is not so efficient compared to hematite, although its importance to atmospheric ice formation will be evaluated in a later section. Highly concentrated aqueous solutions of citric acid can be highly viscous and kinetically limit water uptake.^83,114^ The glass transition temperature at *RH*_i_ = 100% has previously been estimated at 211.8 K (ref. 115) and likely, glassy citric acid is not important in our investigated *T* and *RH*_i_ range.

Feldspar particles coated with xanthan gum nucleated ice *via* deposition ice nucleation at 244 K. Water uptake was not detected at this temperature. At *T* = 256 K, however, water uptake occurred at *RH*_w_ = 91% followed by immersion freezing. A previous study showed that water uptake by xanthan gum at room temperature and *RH*_w_ = 91% led to a growth in particle diameter by a factor of 1.2 compared to its dry diameter when *RH*_w_ = 0%. These are concentrated xanthan gum solutions that are highly viscous and exhibit glass transition temperatures of about 256.8 K and 249.9 K when *RH*_w_ = 11 and 84%, respectively,^89^ seen as the orange line in Fig. 2 extrapolated to *RH*_w_ = 95%. It can be expected that the ability for xanthan gum to take up water is kinetically limited to the left of this glass transition line in Fig. 2. We compare our deposition ice nucleation results with those of onset deposition ice nucleation of feldspar particles without any organic present shown as gray crosses^58^ for particles with a 300 nm electrical mobility diameter and a frozen fraction of about 1 in 10^4^ particles. We also compare with supermicron sized particles, shown as open diamonds.^51^ There is good agreement between the *RH*_i_ where deposition ice nucleation was observed for feldspar particles with and without a xanthan gum coating. This implies that either the ice nucleation efficiency of glassy xanthan gum is similar to feldspar, or the xanthan gum did not affect ice nucleation properties of feldspar if water mobility through the xanthan gum was not limited and could access the feldspar particle surface. Ice nucleation studies using xanthan gum particles alone are certainly necessary to verify the former explanation, however, they were not possible due to time constraints. Diffusion coefficients of water through xanthan gum should also be determined as a function of *RH*_w_ and *T* to elucidate if deposition ice nucleation was due to the glassy xanthan gum surface or the feldspar beneath. In contrast to our deposition ice nucleation results, immersion freezing was observed after water uptake due to the xanthan gum at *RH*_w_ = 96%. Therefore, feldspar nucleated ice in aqueous xanthan gum at water subsaturated conditions. Feldspar immersion freezing has been previously shown to commonly follow a freezing point depression,^40^ meaning that ice nucleation from feldspar in aqueous solution is expected to occur at lower temperature than for particles in pure water. Previous studies on feldspar shown as the solid and dashed gray lines along *RH*_w_ = 100% in Fig. 2 observed ice nucleation in pure water in agreement with our immersion freezing data considering the experimental error in *RH*_i_. The xanthan gum aqueous solution that formed may have been highly dilute. As described later, the immersion freezing data presented here for aqueous solution can be predicted similarly as immersion freezing in pure water using the aqueous solution water activity, *a*_w_, which is equivalent to *RH*_w_ when the solution is in equilibrium with water vapor.^22^

### 3.2 X-ray imaging and NEXAFS spectroscopy of water droplets and ice particles

**Fig. 3:**
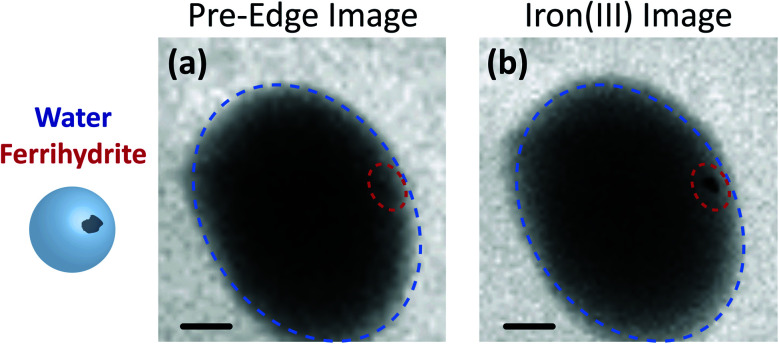
A water droplet imaged with a ferrihydrite particle immersed inside at 236 K. The image in (a) was taken at the iron pre-edge (700.0 eV) and the image in (b) was taken at the resonant energy (709.6 eV) corresponding to iron(iii). The blue and red outlines indicate where the water and ferrihydrite particle are, respectively. The scale bar in both images is 2 μm.

Fig. 3 shows X-ray images acquired at 700.0 and 709.6 eV of a water droplet with a ferrihydrite particle immersed inside at *T* = 236 K and *RH*_w_ = 100%. The droplet is visible at both X-ray energies, however, the ferrihydrite particle is only visible in Fig. 3(b) at 709.6 eV, corresponding to the resonant absorption energy for iron(iii). Ice formation did not occur at this low temperature while the liquid-vapor equilibrium was maintained for hours, demonstrating our ability to well-control thermodynamic conditions in the INXCell. If ice formed heterogeneously elsewhere on the sample, *e.g.* on the silicon nitride substrate or the Pt temperature sensor, the vapor pressure decrease would result in rapid evaporation of the droplet. This did not occur and implies that the substrate and Pt wire did not nucleate ice heterogeneously. In the presence of citric acid, droplets always formed followed by ice nucleation.

**Fig. 4:**
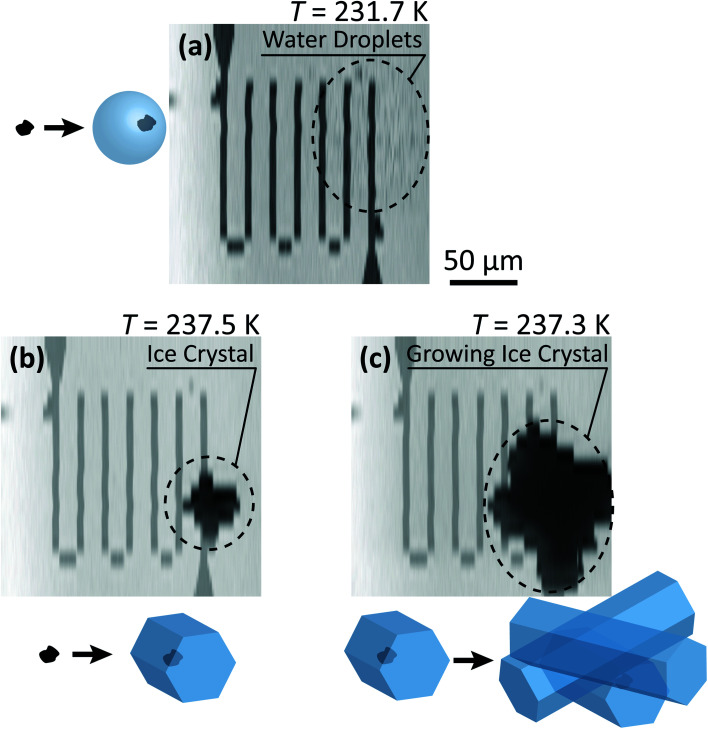
Example of an INXCell experiment in which (a) water droplets formed, following by (b) ice formation (c) and crystal growth on dry deposited ferrihydrite particles. The temperature, *T*, of the sample is indicated above the STXM images. The coarse spatial resolution was necessary to quickly image the nucleated and growing ice crystal.

**Fig. 5:**
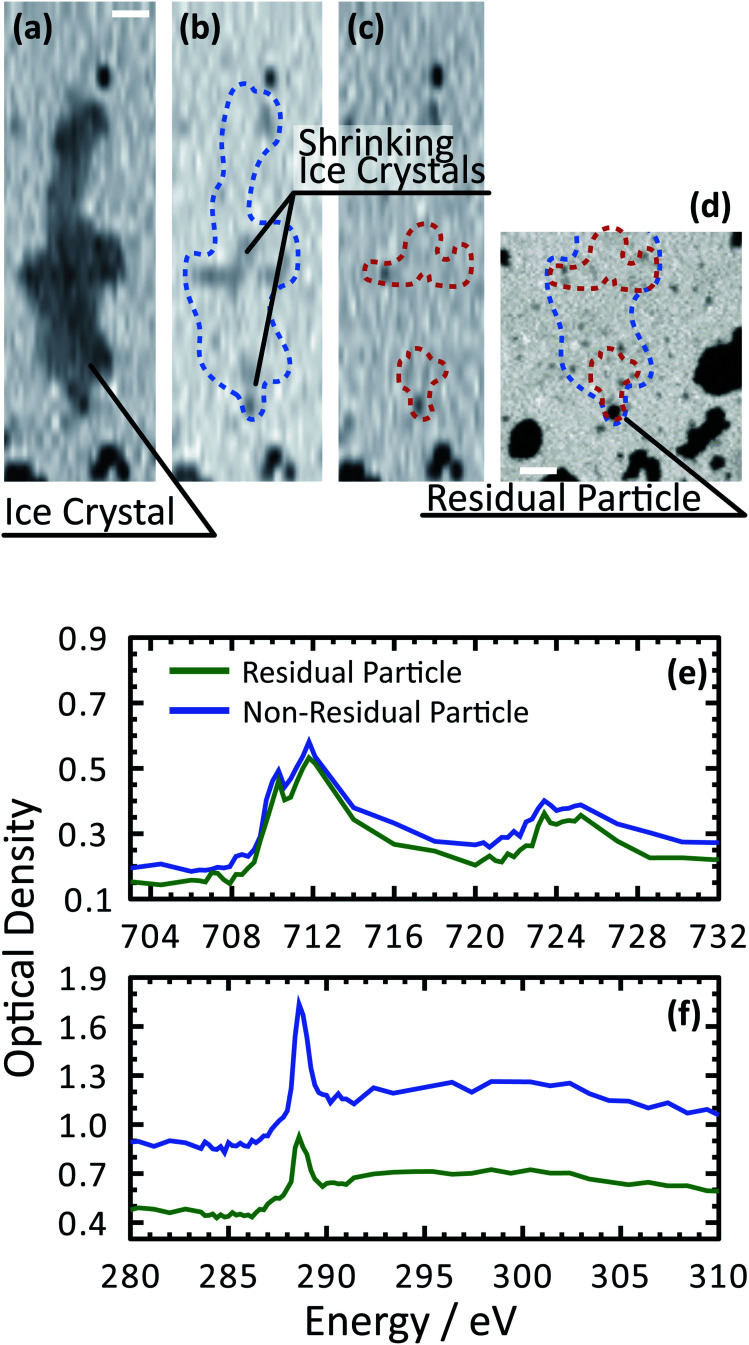
Demonstration of ice nucleation and spectroscopic identification of ferrihydrite particles coated with citric acid. (a)–(c) A sequence of X-ray images at 280 eV showing the last instances of a sublimating ice crystal. The coarse resolution is necessary to quickly image the shrinking crystal. The blue and orange outline indicates the crystal boundaries. (d) An X-ray image at 288.6 eV showing the crystal boundaries, residual particles after sublimation and organic rich particles across the sample. The scale bar is 2 μm for all images. NEXAFS spectra were acquired first (f) at the carbon K-edge and then (e) the iron L_2,3_-edges of a residual particle and a non-ice nucleating particle.

Our ability to detect droplet formation, ice nucleation and identify residual particles from sublimating ice crystals is shown in Fig. 4 and 5. During cooling, X-ray optical density images at a coarse resolution were acquired. Our Pt temperature sensor is seen in Fig. 4(a) along with droplets that formed at 231.7 K. It is important to note that the droplets appeared simultaneously across the surface indicating a uniform water vapor distribution and very low temperature gradient within the dashed circled area in Fig. 4(a). In this particular cycle, ice formed 0.2 K lower in temperature seen in Fig. 4(b). The ice crystal grew significantly after cooling by another 0.2 K seen in Fig. 4(c). After a calibration procedure described above, the ice crystal was subjected to slow sublimation. Fig. 5 shows example images during sublimation for an ice crystal that formed on citric acid coated ferrihydrite. The shrinking ice crystals were imaged with a coarse resolution in Fig. 5(a) and (b). Finally, the ice completely sublimated, as seen in Fig. 5(c), and a high spatial resolution image was acquired, as seen in Fig. 5(d). We obtained NEXAFS spectra at the carbon K-edge and iron L_2,3_-edges shown in Fig. 5(e) and (f), respectively, of the residual particle indicated in the image and a non-residual particle elsewhere on the sample. Both have nearly identical spectral features indicating the presence of ferrihydrite and citric acid. Considering our nanoscale spatial resolution (∼35 nm) and our chemical sensitivity from NEXAFS spectroscopy, we have found no significant difference between these particles. Therefore, it is evident that ice nucleation randomly nucleated on these particles, where larger particles have a greater change to be INPs.

### 3.3 Modelling homogeneous and heterogeneous ice nucleation

Ice formation is well-established in nucleation theory, which is used to derive a freezing rate that is crucial to the nucleation process. Water clusters form with increasing numbers of molecules coinciding with increasing free energy until a potential energy barrier is overcome triggering energy release and thus nucleation followed by bulk crystallization.^116^ The homogeneous ice nucleation rate coefficient, *J*_hom_, with units of cm^−3^ s^−1^ predicts water and aqueous solution droplet freezing in the atmosphere.^22,24,116,117^ The homogeneous ice nucleation rate is *ω*_hom_ = *J*_hom_*V*_d_, where *V*_d_ is the volume of a droplet. A heterogeneous ice nucleating substrate reduces the energy barrier for ice nucleation, and the heterogeneous ice nucleation rate coefficient is *J*_het_, with units of cm^−2^ s^−1^. From this, the heterogeneous ice nucleation rate, *ω*_het_, scales with the particle surface area, *A*_p_, where *ω*_het_ = *J*_het_*A*_p_. Immersion freezing from laboratory generated particles, from ambient particles and in atmospheric cloud models has been successfully described using the water activity, *a*_w_, of the bulk solution around the immersed particle,^39–41,118,119^ being either pure water or aqueous solution. The *a*_w_-based immersion freezing model (ABIFM) quantifies *J*_het_ over a range of atmospherically relevant *T* and *RH*_w_ using *a*_w_ and shown to be valid thus far for every investigated organic, biogenic and mineral type of particles and for every organic or inorganic solute.^39,40,119^ Due to its vast applicability for heterogeneous freezing both at and below water saturation, we use the ABIFM to derive *J*_het_ for immersion freezing due to ferrihydrite with and without coatings of citric acid, as well as immersion freezing due to feldspar coated with xanthan gum. *J*_het_ for immersion freezing of feldspar in aqueous solution was previously derived,^40^ although this was not tested with feldspar in xanthan gum solutions. A *a*_w_-based deposition ice nucleation model (ABDINM) was not previously considered, because aqueous solution is thought not to be involved in deposition ice nucleation. Despite this, we use the ABIFM and ABDINM as convenient and reliable functional forms of *J*_het_ for model implementation described below.

For immersion freezing, *J*_het,im_, as a function of the water activity criterion, Δ*a*_w_,^39,40^ is parameterized as

1 *J*_het,im_ = *m*_im_Δ*a*_w_(*a*_w_,*T*) + *c*_im_,

where *m*_im_ and *c*_im_ are parameters specific to either ferrihydrite or feldspar. The term, Δ*a*_w_, is the difference between solution water activity and the water activity along the ice melting point line at constant *T*, or

2 Δ*a*_w_(*a*_w_,*T*) = *a*_w_ − *a*^i^_w_(*T*),

where *a*_w_ is assumed equal to *RH*_w_ the particle is exposed to in the INXCell, and *a*^i^_w_(*T*) is the ice–liquid equilibrium curve.^22,120^ As previously mentioned, a major advantage of eqn (1) is that it is independent of the nature of the solute and so is applicable to aqueous solutions of both xanthan gum and citric acid. In other words, a single value of Δ*a*_w_ defines a unique point in the *T versus a*_w_ phase diagram where *J*_het,im_ is constant and independent of any solute. Eqn (1) therefore relates heterogeneous ice nucleation kinetics directly to thermodynamics.^39,40^

To represent deposition ice nucleation, we have used the same functional form as eqn (1) where

3 *J*_het,dep_ = *m*_dep_Δ*a*_w_(*a*_w_,*T*) + *c*_dep_,

and *m*_dep_ and *c*_dep_ are parameters different from those for immersion freezing. We have expanded the stochastic freezing model (SFM) presented in Alpert and Knopf^40^ to predict the freezing probability, *P*_frz_, of a particle due to immersion freezing, deposition ice nucleation and homogeneous freezing as4
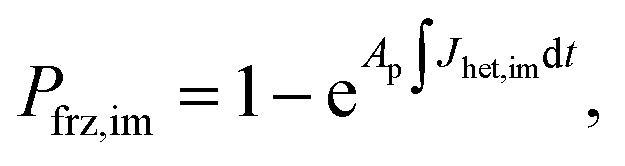
5
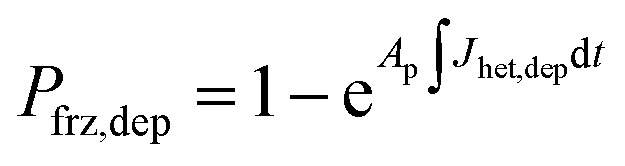
and6
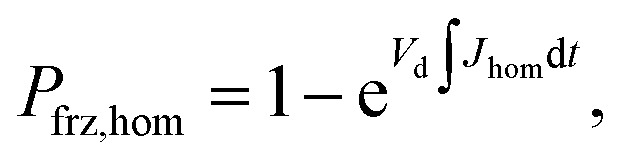
respectively. Eqn (1)–(3) are used in eqn (4) and (5), while *J*_hom_ is taken from literature^22,120^ and used in eqn (6). The integral with respect to time, *t*, is included in eqn (4)–(6) to account for a variable cooling rate, *i.e. T*(*t*), of the sample material. The updated SFM presented here simulates freezing by single particles,^40^ each having a unique *A*_p_. The uncertainty in quantifying *A*_p_ was previously shown to significantly impact freezing results.^40^ Therefore, we derived *A*_p_ and the distribution of particles sizes and total surface area on a sample from measurements of particle sizes on the sample estimated from STXM images. These were used to produce a surface area probability distribution from which the SFM randomly sampled *A*_p_ for one simulation. The temperature in the SFM decreases continuously and after a small temperature step, Δ*T* = 0.2 K, freezing of a particle is sampled from a binomial distribution as a Bernoulli trial using a probability parameter from eqn (4)–(6). A success is considered an ice nucleation event and a single simulation is finished as soon as a freezing event is sampled. We then use a Monte Carlo method by repeating the simulation 10^5^ times to determine the average conditions of *T*, *RH*_w_ and *RH*_i_ at which ice occurs and their uncertainty, which is derived from the multiple simulations and from Poisson statistics at 99.9% confidence following Alpert and Knopf.^40^ It is important to note that each simulation run will sample a new particle population from the measured size distribution to account for the uncertainty in surface area. Finally, parameters *m*_im_, *m*_dep_, *c*_im_ and *c*_dep_ were varied to best fit the average thermodynamic conditions at which ice was observed to form, while also matching the standard deviation from multiple cooling cycles.

One of the main goals of modelling ice nucleation using the SFM was to reproduce our results and uncertainties in *RH*_i_ and *T* at which freezing was observed. In addition, we evaluated the variability in *RH*_i_ and *T* that could be attributed to random freezing. As previously stated, only the first ice nucleation event on a sample with many other particles was observed and due to time constraints, we were limited on the number of repeated cooling cycles (see Table 1) that could be performed. Therefore, the SFM was employed to assess the statistical probability of a single ice nucleation event and compare it to the *RH*_i_ and *T* variability.

**Fig. 6:**
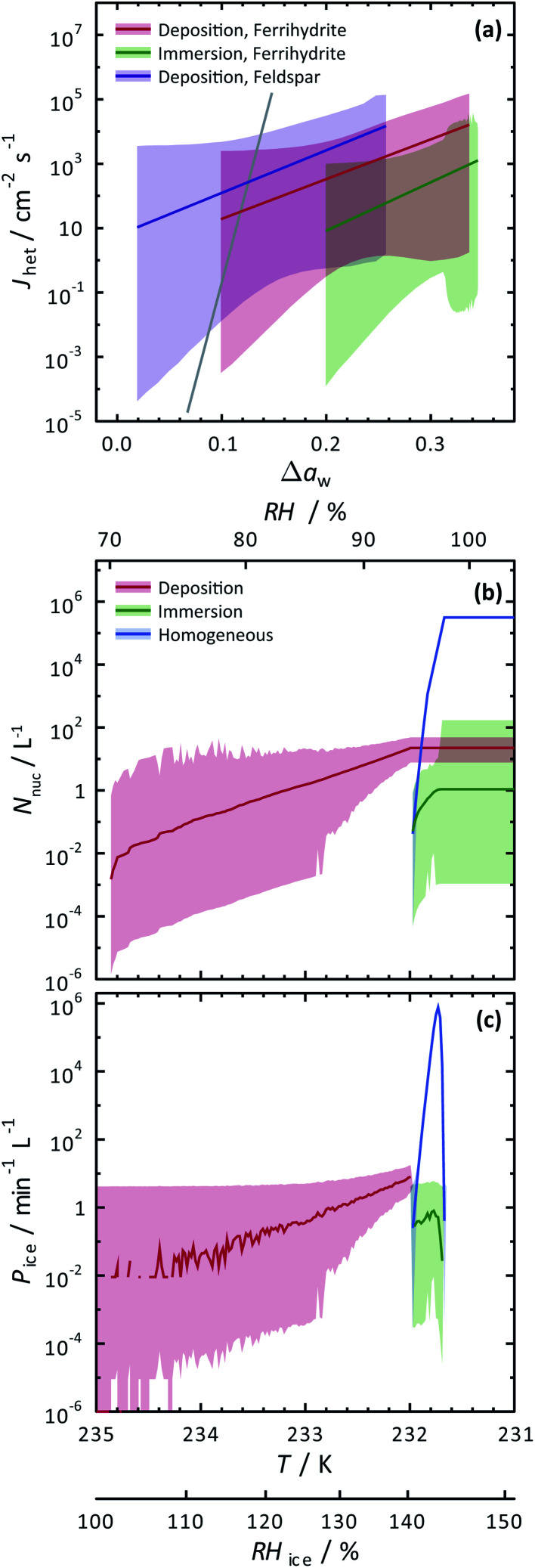
Calculated heterogeneous ice nucleation rate coefficients, *J*_het_, ice nucleation events, *N*_nuc_, and ice particle production rates, *P*_ice_, are shown. (a) *J*_het_ parameterizations and their certainty at 0.999 confidence are shown as the solid colored lines and shadings. The solid gray line is for immersion freezing due to feldspar from Alpert and Knopf.^40^ Modelled ice nucleation accounting for deposition ice nucleation, immersion freezing and homogeneous ice nucleation is shown in (b) and (c) from an aerosol population having ferrihydrite and non-ferrihydrite particles. Lognormal distributions with two modes were used with parameters *N*_1_ = 285 cm^−3^, *μ*_1_ = 0.05 μm, *σ*_1_ = 1.2, *N*_2_ = 15 cm^−3^, *μ*_2_ = 0.9 μm and *σ*_2_ = 0.5 for non-ferrihydrite particles and *N*_3_ = 14 cm^−3^, *μ*_3_ = 0.05 μm, *σ*_3_ = 1.2, *N*_4_ = 0.8 cm^−3^, *μ*_4_ = 0.9 μm and *σ*_4_ = 0.5 for ferrihydrite particles.

The SFM was able to reproduce our measured conditions as seen in Fig. 2. Also, the standard deviation of modeled *RH*_i_ derived entirely from stochastic variability was comparable to the *RH*_i_ measurement uncertainty. Fitted values were *m*_dep_ = 12.3525, *c*_dep_ = 0.0516, *m*_im_ = 15.0469 and *c*_im_ = −2.0906 for ferrihydrite particles, and *m*_dep_ = 13.2251 and *c*_dep_ = 0.7716 for feldspar particles. Parameters for feldspar particle immersion freezing are *m*_im,feld_ = 122.83 and *c*_im,feld_ = −12.98 from Alpert and Knopf.^40^ New values of *J*_het,dep_ and *J*_het,im_ for both ferrihydrite and feldspar and their error are shown in Fig. 6(a). The SFM was run for deposition, immersion and homogeneous freezing simultaneously, where the latter two were allowed only after water uptake, which was observed for ferrihydrite at about *RH*_w_ = 94.5%^+4.7^_−4.5_. Particles having citric acid or particles with xanthan gum above the glass transition (orange line in Fig. 2) were modelled to always have water and nucleate ice *via* immersion freezing, while deposition ice nucleation was not allowed. We reiterate that deposition ice nucleation on ferrihydrite without citric acid was observed in 7 out of 8 experiments around *T* = 232 K (see Table 1), while immersion freezing occurred once. Our model similarly predicted that deposition ice nucleation would occur about 85% of the time in competition with immersion freezing and homogeneous ice nucleation. At around *T* = 232 K, the SFM predicted that deposition, immersion and homogeneous freezing would occur on ferrihydrite at 75%, 5% and 20%, respectively. In addition, citric acid/ferrihydrite particles formed ice around conditions expected for homogeneous freezing. Although we did observe residual particles after freezing for both ferrihydrite particles and citric acid coated ferrihydrite particles, there remains a possibility that homogeneous ice nucleation may have occurred instead when considering the uncertainty in our observations, *i.e.* the error bars for immersion freezing overlap with expected homogeneous freezing temperatures. We find that immersion freezing may not be a competing way of nucleating ice for ferrihydrite particles. Instead, we claim that deposition ice nucleation can be important for heterogeneous ice nucleation if water uptake does not occur.

The SFM prediction of deposition ice nucleation around *T* = 244 K on mixed feldspar/xanthan gum particles is in good agreement with observations. We find that at subsaturated conditions, deposition ice nucleation may be important for atmospheric ice nucleation from feldspar particles. Deposition ice nucleation was predicted to occur 100% of the time, meaning that *RH*_w_ = 100% was not reached to allow for droplet formation followed by immersion freezing. This is in agreement with our ice nucleation and water uptake observations. Around *T* = 255 K, we observed water uptake and immersion freezing below water saturation. Note that Table 1 details the number of repeat cooling cycles for each experiment. The SFM predicted immersion freezing of feldspar is in agreement with our observations, and verified the parameterization from Alpert and Knopf.^40^ It is important to note that our error bar derived from temperature and humidity uncertainty is much larger than the error from our model derived from stochastic freezing. This is not a disagreement since the SFM does not consider any temperature error, and only implies that stochastic error may not contribute very significantly to the total error in this case.

### 3.4 Atmospheric importance of ferrihydrite

To evaluate the importance of deposition ice nucleation and immersion freezing due to ferrihydrite particles, or homogeneous freezing, we applied the SFM to a hypothetical aerosol size distribution representative of the concentration of fine particles and coarse mode airborne dust particles.^42,121,122^ In this exercise, dust particles were arbitrarily set to 5% of the total particles. Using *J*_het,dep_ and *J*_het,im_ for ferrihydrite from Fig. 6(a), and allowing for multiple freezing events we modelled freezing and tracked the ice particle production rates and concentration of ice particles due to all three ways of ice nucleation. Note, that using the SFM in this way does not accurately represent atmospheric ice nucleation as it lacks air mass trajectories, crystal growth, water vapor depletion, cloud structure and other thermodynamic and meteorological processes that impact cloud microphysics. However, it does establish the potential importance of ferrihydrite to atmospheric ice nucleation. Importantly, it also evaluates the competition between immersion, deposition and homogeneous freezing in determining the range of conditions that one may be more important than the other. Further parcel or cloud resolving models would be necessary to give estimates that are more accurate.

Fig. 6(b) and (c) show the results of the SFM employing our representative size distribution with a cooling rate of 0.1 K min^−1^. The number of ice nucleation events, *N*_nuc_, due to deposition ice nucleation (red lines) reach 10^−2^ L^−1^ of air before immersion or homogeneous freezing occurred at a rate, *P*_i_ < 10^−2^ min^−1^ L^−1^ near *RH*_i_ = 100%. These initial freezing events are due to the largest coarse mode particles that have more surface area and thus a higher probability to freeze. At a maximum, *N*_nuc_ = 21 L^−1^ and *P*_i_ = 8 min^−1^ L^−1^ from deposition ice nucleation alone. Water uptake was modelled at *RH*_w_ = 94.5%, which was similar to observations for ferrihydrite particles. After water uptake, deposition ice nucleation was not possible and immersion and homogeneous freezing was allowed. Homogeneous freezing contributed far more to the total *N*_nuc_ toward the end of the model run than immersion freezing shown in Fig. 6(b). In order to assign an uncertainty to these model predictions, we consider a hypothetical instrument counting ice particles similar to a continuous flow diffusion chamber (CFDC) that samples 1 L min^−1^ of air with a 10 s resident time and using a 10 min averaging interval. These CFDC instruments are routinely deployed in aircraft studies for measuring INP concentrations.^42,123,124^ Again, using Poisson statistics at 99.9% confidence, we derive upper and lower fiducial limits^125^ of *N*_nuc_ and *P*_i_ seen as the shaded areas. Ambient INPs and ice crystal concentrations are observed from 10^−1^ to 10^3^ L^−1^ in atmospheric clouds, however these numbers are highly uncertain in general and not well-known for cirrus clouds.^28^ We find that low numbers of ice crystals in clouds could be due to deposition ice nucleation from ferrihydrite in dust, but also we claim that these low numbers, and thus low sampling statistics, are the leading cause of the great uncertainty associated with predictions. Reducing the uncertainties would require high volume sampling and long particle residence times in ice nucleation instrumentation to detect high numbers of INPs, which poses a significant experimental challenge. However, despite low numbers of ice nucleation events, careful evaluation of uncertainties, as done here, can still yield increased understanding of ice nucleating particle chemistry and physics. We have demonstrated that ferrihydrite particles have the ability to nucleate ice and should be considered in future deposition ice nucleation studies and derived quantitative values of *J*_het_ and corresponding uncertainties following water activity to predict ice particle production.

Our INXCell and SFM are also well-suited for measuring and modelling the ice nucleation ability of field collected particles in combination with their chemical morphology. Due to the wide variability of particle types, *in situ* identification and composition mapping of organic coatings and metal oxidation states of atmospheric INPs will give valuable insights on the nature of ice nucleation. In doing so, future studies will be able to discriminate *in situ* INP types and their composition with discrimination of immersion freezing and deposition ice nucleation. We recommend further investigation on ambient particles using our new INXCell and newly developed SFM. Furthermore, experiments on complex laboratory generated particles using the INXCell would greatly benefit from spectroscopic and composition mapping data. This could be applied in future ice nucleation studies, *e.g.* on both fully or partially coated particles.

## 4 Conclusions

Ice nucleation due to ferrihydrite particles with and without citric acid, as well as feldspar with xanthan gum, was observed in a new instrument, referred to as the INXCell, that combines X-ray spectro-microscopy, a humidified environmental chamber and a cryogenic cold stage. This work showed that ice nucleation on single particles could be paired with *in situ* environmental spectroscopy. In this way, the detection of specific chemical components on and inside single particles could be distinguished such as inorganic material, organic material, iron, and corresponding carbon bonding functionalities and oxidation state. In particular, single ice nucleating ferrihydrite particles were identified through X-ray images and NEXAFS spectroscopy. Ferrihydrite nucleated ice at significantly lower values of *RH*_i_ than expected for homogeneous ice nucleation. This occurred only when water uptake was completely avoided. When water uptake did occur, ice nucleation occurred at conditions close to homogeneous ice nucleation. One surprising finding was that even at the same humidity conditions, repeat cycles were performed that either did and did not result in observed water uptake. Therefore, heterogeneous ice nucleation *via* deposition ice nucleation from the water vapor phase was in competition with water uptake onto ferrihydrite particles. This was possibly due to the stochastic nature of heterogeneous ice nucleation, *i.e.* if ice formation did not occur by chance, then water uptake would eliminate any further chance of deposition ice nucleation. We also observed single ferrihydrite particles immersed inside of supercooled water droplets without any indication of ice nucleation and rapid crystallization. Feldspar deposition ice nucleation in the presence of xanthan gum was observed and was in agreement with previous ice nucleation results at water subsaturated conditions for feldspar ice nucleation studies without xanthan gum. Although xanthan gum is hygroscopic, no water uptake was observed on the mixed particles, likely due to the xanthan gum being a glass at the humidity and temperature at which ice nucleation was observed.

We quantified ice nucleation rate coefficients using observation of *T* and *RH*_i_ at which ice formed using our newly developed model, the SFM, and carefully determined corresponding uncertainties. This was an extension of our previously developed immersion freezing model using a Monte Carlo technique.^40^ This model uses a water activity based description for quantifying freezing kinetics (*J*_het_) and thus, independent of any solute type present and applicable for both water saturated and water subsaturated conditions. The SFM could reproduce our observations of ice nucleation due to ferrihydrite and feldspar with and without citric acid or xanthan gum aqueous solution and within our experimental error. The range of *RH*_i_ at which particle froze predicted by the SFM was almost identical to the scatter in our observations. Since the SFM variability is entirely based on a stochastic freezing process, we found that stochastic freezing was the major source of error for deposition ice nucleation and immersion freezing due to ferrihydrite particles and deposition ice nucleation due to feldspar particles. On the other hand, the variability of *RH*_i_ predicted by the SFM for immersion freezing caused by feldspar was much smaller and thus, experimental temperature error was likely the major source of variability. The fact that stochastic freezing has the ability to explain our data scatter and error gives evidence that heterogeneous ice nucleation due to ferrihydrite and feldspar is dominated by stochastic freezing and validated the use of a nucleation rate coefficient following nucleation theory.

We have also investigated the potential importance of ferrihydrite as an atmospheric ice nucleating particle using our SFM, considering the competition between immersion freezing, deposition ice nucleation and homogeneous ice nucleation. Concentrations of ice particles from deposition ice nucleation were predicted on the order of 10^1^ L^−1^ when *RH*_i_ increased up to 145%. These crystal numbers are typical for cirrus cloud formation, and thus, ferrihydrite could potentially be important for atmospheric ice production if water uptake is avoided. Immersion freezing insignificantly contributed to the total ice particle concentration compared to homogeneous freezing, and therefore, immersion freezing of ferrihydrite is concluded to not be important for atmospheric ice production.

This work could only be possible using our new INXCell, which features a platinum resistive temperature sensor that was lithographically patterned onto the sample surface and a novel cryogenic cooling solution. This ensured a high precision measurement of the sample temperature as low as 230 K, demonstrated in this study. The major challenge to overcome was to ensure that the coldest area of the gas flow path throughout the inside of the entire INXCell construction was located at the X-ray transparent silicon nitride membrane where particles sit, and co-located where the temperature of the particles was measured. This yielded unprecedented accuracy and control of temperature and thus humidity. Inside the cell, this means that maximum relative humidity conditions are central, localized, predictable and uniform across a defined sample area. To our knowledge, measurement and equilibration of pure water droplets, *i.e.* at 100% relative humidity, with and without particle immersed using STXM/NEXAFS has never been done until now. The INXCell is advantageous for future studies of, *e.g.* phase transitions, chemical reactions, or multiphase chemistry, but potentially in other disciplines such as electrochemistry^106^ for fuel cell performance,^126,127^ involving the aqueous phase of liquid water where water saturation is maintained for long time scales.

## Data availability

All data are publicly available online at https://doi.org/10.5281/zenodo.6034243. This includes schematic drawings of the order selecting aperture.

## Code availability

All codes are publicly available online at https://doi.org/10.5281/zenodo.6034243.

## Author contributions

P. A. A. wrote the manuscript. P. A. A., B. W., C. P. and M. A. conceptualized and planned the study. P. A. A. planned and conducted STXM/NEXAFS experiments supervised by B. W., C. P. and M. A., A. B., S. Y., H. Y. and K. K. also conducted STXM/NEXAFS experiments. P. A. A. and S. F. designed the INXCell supervised by B. W., S. F. performed lithography. Z. L. performed sputter coating and characterized the temperature sensors. P. A. A. conducted STXM/NEXAFS data analysis and interpretation of results. P. A. A. wrote and developed the SFM. All co-authors discussed the results and commented on the manuscript.

## Conflicts of interest

We declare no conflict of interest.

## Supplementary Material

EA-002-D1EA00077B-s001
