# Supplementary material for: Ice nucleation imaged with X-ray spectro-microscopy
Source: Environ Sci Atmos. 2022 Feb 7;2(3):335–51. doi: 10.1039/d1ea00077b (PMC9119033; doi:10.1039/d1ea00077b)
Supplement: EA-002-D1EA00077B-s001 [file EA-002-D1EA00077B-s001.pdf]

Electronic Supplementary Material (ESI) for Environmental Science: Atmospheres.
This journal is © The Royal Society of Chemistry 2022

Ferrihydrite: An Excellent Ice Nucleation Particle

Environmental Science: Atmospheres

The formation of ice crystals in the upper atmosphere is a key step in the water cycle

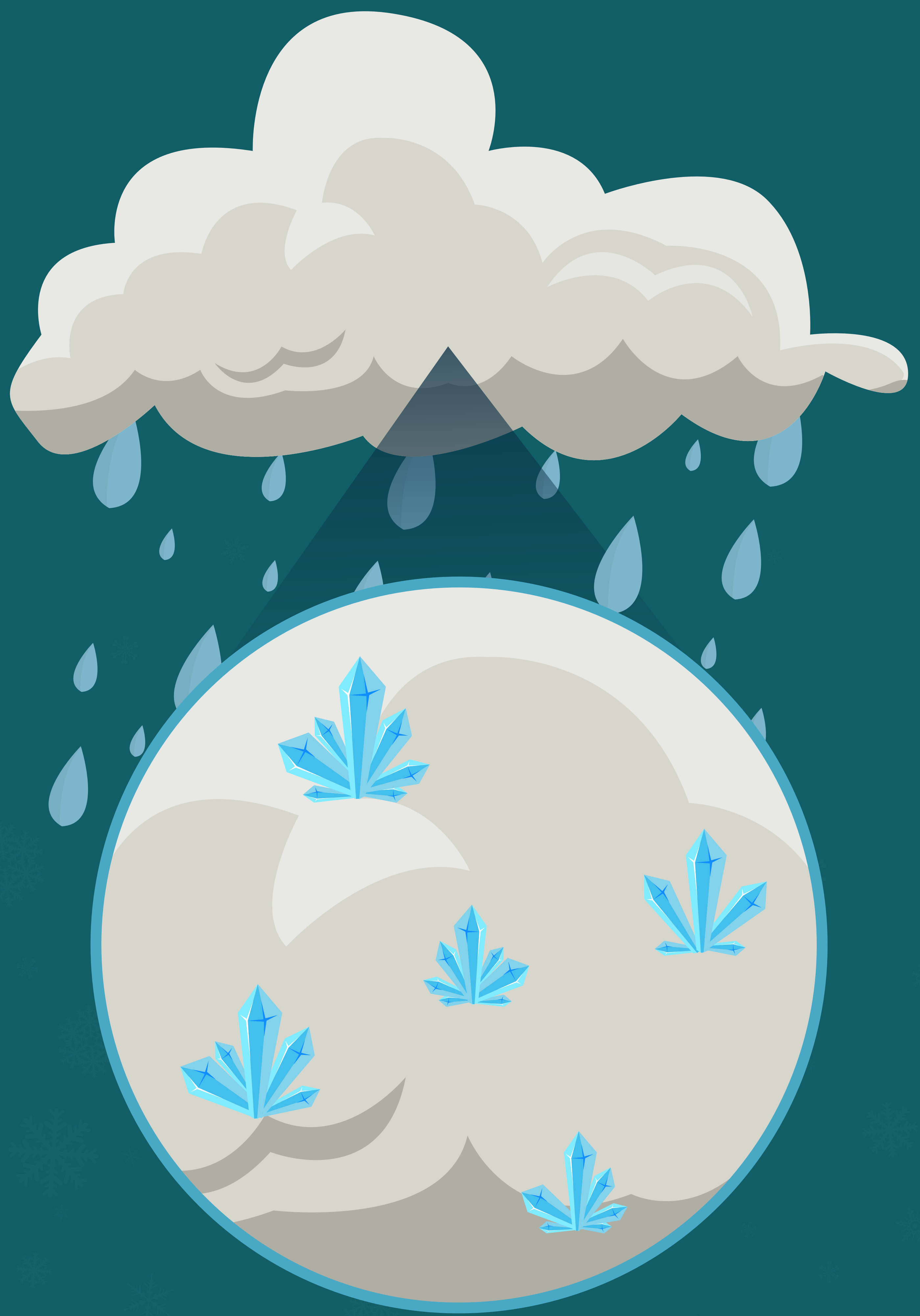

However, little is known about the chemical components catalyzing such ice formation on nanometer scales

Can atmospheric particles catalyzing ice nucleation be used for precipitation and weather prediction?

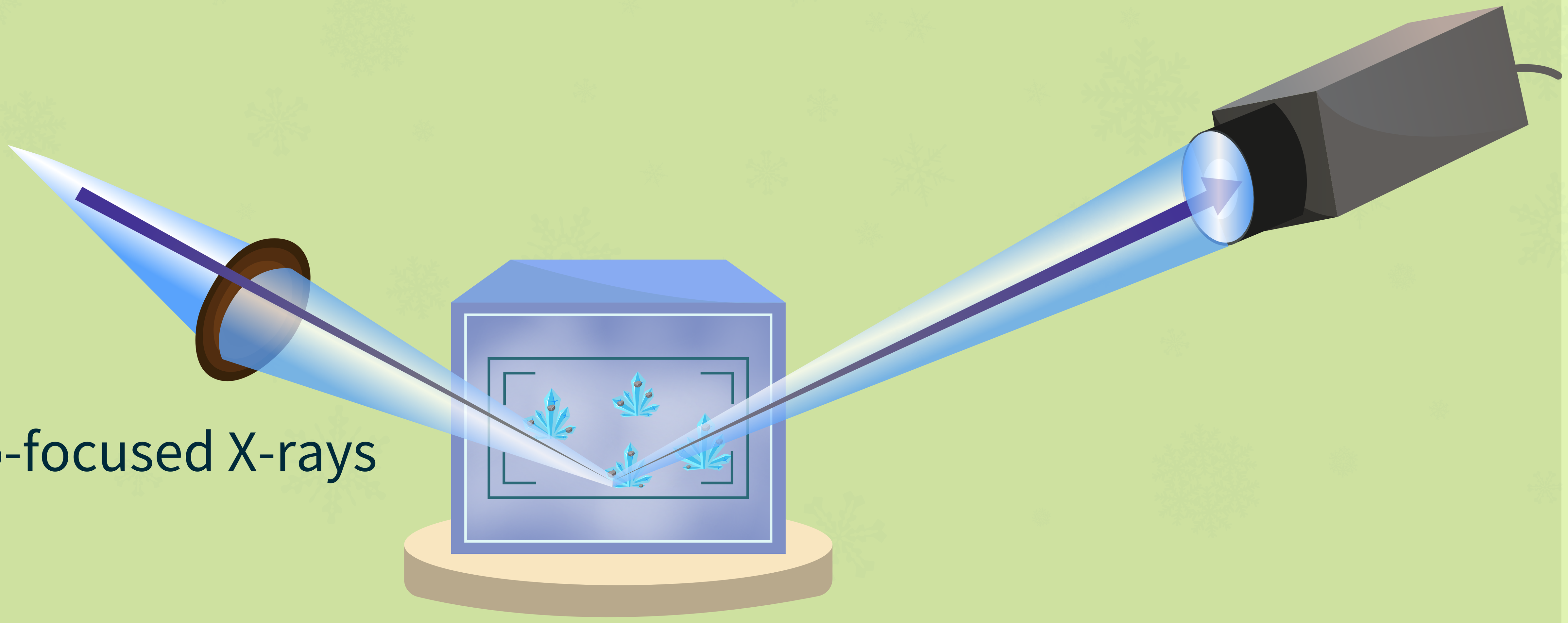

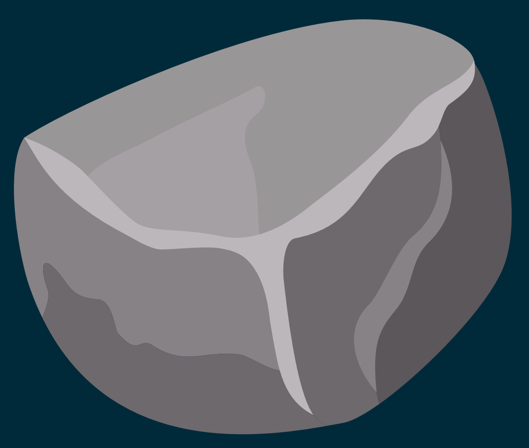

Ferrihydrite, an iron-containing particle, catalyzes ice nucleation

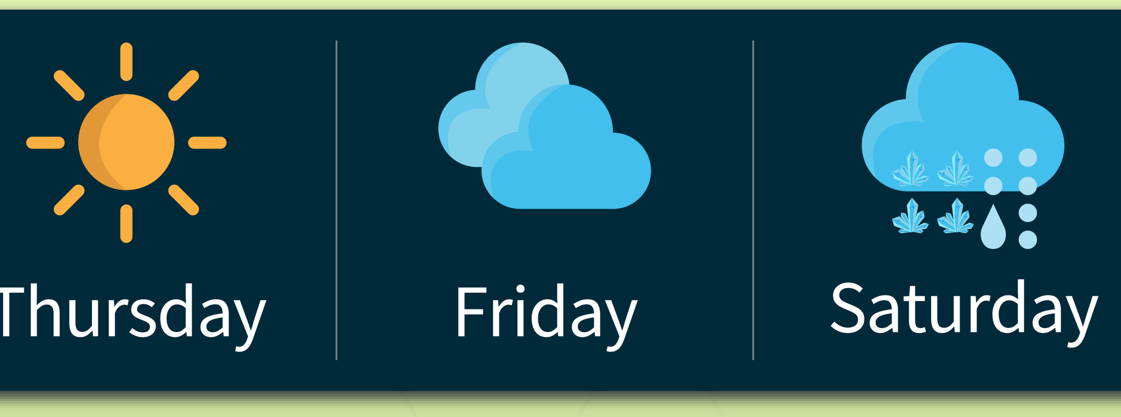

Ferrihydrite levels can predict ice crystals in clouds

Ferrihydrite aids ice formation and can be used to model cloud behavior aimed at better climate and weather prediction

Ice nucleation imaged with X-ray spectro-microscopy

Alpert et al. (2022) | Environmental Science: Atmospheres | DOI: 10.1039/D1EA00077B

ROYAL SOCIETY OF CHEMISTRY
